# Tissue eosinophilia suggests dupilumab treatment response in chronic rhinosinusitis with nasal polyps (CRSwNP)

**DOI:** 10.1007/s00405-026-10187-y

**Published:** 2026-04-29

**Authors:** Patrick Huber, Till Braunschweig, Hanna Frankenberger, Clemens Stihl, Moritz Gröger, Donata Gellrich, Manuel Lasch

**Affiliations:** 1 Department of Oto-Rhino-Laryngology, Head and Neck Surgery, Ludwig Maximilian University of Munich, Marchioninistraße 15, Munich, 81377 Germany; 2 Institute of Pathology, Ludwig Maximilian University of Munich, Munich, Germany

**Keywords:** CRSwNP, Dupilumab, Type-2 inflammation, Biomarkerbiomarker, Tissue eosinophilia

## Abstract

**Background:**

Chronic rhinosinusitis with nasal polyps (CRSwNP) is predominantly driven by type 2 inflammation and frequently persists despite standard therapy. Dupilumab has proven efficacy, yet robust predictors of long-term response remain insufficiently defined.

**Methods:**

In this monocentric, retrospective analysis, 163 adults with CRSwNP treated with dupilumab for ≥ 24 months were assessed at a tertiary referral center. Outcome parameters included nasal polyp score (NPS), sinonasal quality of life (SNOT-22), and olfactory function (SSIT). Treatment response was classified according to EUFOREA 2023 criteria. Archived polyp tissue from 72 patients obtained prior to Dupilumab treatment underwent histological reassessment, with eosinophilic infiltration quantified using absolute thresholds (Lee et al.: ≤10, 11–69, ≥ 70 eosinophils/HPF) and relative thresholds (Yang et al.: >20%).

**Results:**

Dupilumab led to marked and sustained clinical improvements across the cohort (NPS − 4.6 ± 2.1; SNOT-22 − 44.1 ± 18.5; SSIT + 4.3 ± 2.9 at 24 months). Outcomes were independent of demographic factors, asthma, NERD, allergen sensitization, serum IgE, or blood eosinophils. In contrast, higher tissue eosinophilia correlated with enhanced short-term responses. Patients with > 20% eosinophils showed significantly greater SNOT-22 reduction at 12 months (*p* < 0.05). Stratification by ≥ 70 eosinophils/HPF and Yang’s classification (0–10, 11–69, ≥ 70) consistently showed superior trajectories in high-grade eosinophilia. EUFOREA response rates were highest in these subgroups, with no non-responders observed.

**Conclusion:**

Dupilumab achieved durable benefit across all patient subgroups, while elevated tissue eosinophilia predicted faster and more pronounced short-term improvements. Systematic eosinophil quantification may refine patient stratification and optimize biologic therapy allocation in CRSwNP.

## Introduction

Chronic rhinosinusitis with nasal polyps (CRSwNP) is a persistent inflammatory condition of the nasal and paranasal sinuses, defined by the presence of at least two symptoms, such as nasal obstruction, rhinorrhea, facial pain or pressure, and hyposmia, lasting for ≥ 12 weeks [1, 2]. Affecting approximately 2–6% of the population [3, 4], CRSwNP imposes a considerable socio-economic burden due to its chronic nature and high recurrence rates, and typical onset between 40 and 60 years of age [5, 6].

Recent advances in chronic rhinosinusitis (CRS) research have led to an endotype-driven classification system, as outlined in the European Position Paper on Rhinosinusitis and Nasal Polyps 2020 (EPOS 2020) [4]. In Western populations, up to 85% of CRSwNP cases are associated with type 2 inflammation [7]. This eosinophil-dominant endotype is strongly linked to comorbid conditions such as asthma and allergic rhinitis and is prone to recurrence despite standard treatments, including topical or systemic corticosteroids and endoscopic sinus surgery (ESS) [8].

Type 2 inflammation in CRSwNP is driven by a dysregulated immune response, characterized by the overexpression of key cytokines such as interleukin (IL)-4, IL-5, and IL-13, along with increased activation of innate lymphoid cells (ILC2), macrophages, and mast cells [9]. Eosinophils play a pivotal role in disease pathogenesis by releasing pro-inflammatory mediators, interacting with other immune cells, and promoting tissue remodeling. These processes perpetuate inflammation, tissue damage, and promote nasal polyp formation [10].

This deeper understanding of type 2 inflammation has enabled the development of biologic agents selectively targeting its key cytokines and mediators. In Germany, three biologics are currently approved as add-on therapies for uncontrolled CRSwNP. The first and most frequently prescribed biologic is dupilumab, which received regulatory approval in late 2019 [11], followed by omalizumab and mepolizumab [12]. Dupilumab specifically binds to the IL-4 receptor alpha subunit, a shared component of both the heterodimeric IL-4 and IL-13 receptor complexes, thereby blocking essential signaling pathways of type 2 inflammation [13].

Multiple studies, including those conducted by our group and others, have provided substantial evidence supporting the real-world efficacy of dupilumab in the management of CRSwNP [14–17]. However, treatment responses vary among individuals. In our multicenter study, after three years of therapy, 76.1% of patients were classified as good-to-excellent responders, while 23.9% were categorized as poor-to-moderate responders; notably, no patient met the criteria for non-response [14] according to the EUFOREA 2023 update [18] aligning with other published real-word data [19].

Given the high cost of dupilumab [20], optimizing patient selection and refining indication criteria remain crucial to ensure efficient resource allocation. Several studies have suggested potential predictive biomarkers: elevated eosinophil cationic protein (ECP) levels [21–23], total serum IgE [22], and higher baseline blood eosinophil counts, which were recently linked to greater reductions in nasal polyp score after six months of therapy [23], Collectively, these findings underscore the potential of accessible blood-based markers to guide patient stratification and predict treatment benefit. This study aims to further explore whether specific patient characteristics, clinical traits, or biomarkers can reliably predict treatment response to dupilumab in CRSwNP, thereby improving patient selection for biologic therapy.

## Methods

### Study population

This study was designed as a monocentric, retrospective analysis of real-world data conducted at a single tertiary referral center in Germany. A total of 163 adult patients (≥ 18 years) with CRSwNP were included if they showed insufficient disease control and fulfilled EPOS2020 criteria for biologic therapy. All enrolled patients commenced dupilumab as their first-line biologic add-on treatment, while continuing standard intranasal corticosteroid therapy. Only individuals with a minimum follow-up period of 24 months following treatment initiation (as of July 2025) were eligible.

The study adhered to the principles of the Declaration of Helsinki (1976) and received approval from the local institutional ethics committee and data protection authority under project number 22–0802. Informed written consent was obtained from all participants for data collection and study participation.

### Treatment

Dupilumab (Dupixent^®^, Sanofi S.A.) was administered as a 300 mg subcutaneous injection, self-administered by patients in accordance with the manufacturer’s instructions. The standard dosing regimen was once every two weeks (Q2W). In selected patients demonstrating a good-to-excellent clinical response, the dosing interval was tapered on an individual basis. This approach was guided by evidence supporting extended interdose intervals, as demonstrated in the LIBERTY NP SINUS-52 trial [24].

### Data collection

Prior to initiating dupilumab therapy, a comprehensive medical history was obtained for each patient. This included documentation of prior endoscopic sinus surgeries, use of systemic oral corticosteroids (OCS) prior to biologic initiation and the presence of type 2 inflammatory comorbidities such as patient-reported asthma, allergic rhinitis, urticaria, and atopic dermatitis.

Clinical assessments were performed at baseline and subsequently at regular three-month intervals. For clarity and consistency in data presentation, outcomes are reported at standardized timepoints of 6-, 12- and 24-months post-treatment initiation.

These assessments encompassed both subjective and objective outcome measures, as well as relevant biomarkers such as peripheral blood eosinophil counts. Subjective disease burden was captured using a visual analogue scale (VAS) for global disease impact (0–10 cm), and disease-specific quality of life was evaluated using the Sinonasal Outcome Test-22 (SNOT-22). Objective assessments included the endoscopic Nasal Polyp Score (NPS) as defined by Gevaert and van Zele (0–4 per side, total 0–8) [25], and psychophysical olfactory testing performed using either the Sniffin’ Sticks 12-item (SSIT-12) or the Brief Smell Identification Test (B-SIT). To facilitate comparison, olfactory test scores were normalized to a 0–12 scale (0–6 anosmia, 7–10 hyposmia, 11–12 normosmia) according to Lawton et al. [26].

Follow-up adherence was maintained as closely as possible, with a ± 1 month margin allowed for scheduling variability. Additionally, treatment-related parameters such as adverse events, ongoing OCS use, need for revision endoscopic sinus surgery (ESS), and modifications in dosing intervals were recorded. Treatment response at each interval was categorized according to the EPOS/EUFOREA 2023 consensus into no response, poor-to-moderate response, or good-to-excellent response [18].

### Histological analysis

**Fig. 1:**
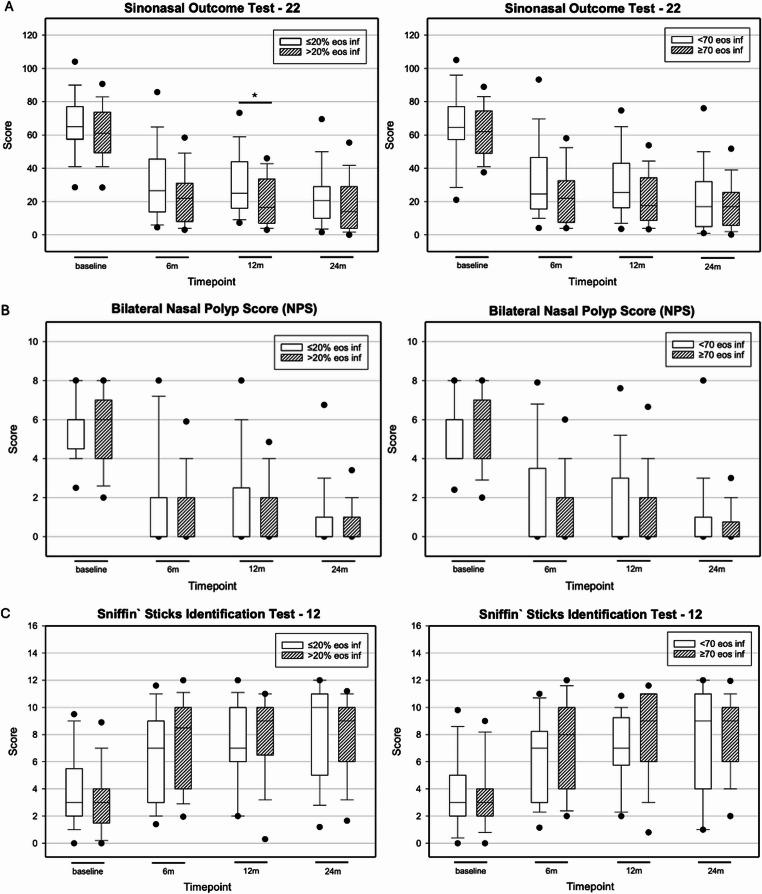
Different patterns of inflammatory cell infiltration in sinus mucosa. (A) Sparse infiltration predominantly composed of lymphocytes (L) and plasma cells (P) with few eosinophils (low cell density per high-power field [HPF], low eosinophil proportion). (B) Dense infiltration of lymphocytes and plasma cells with few eosinophils (high overall cell density per HPF, but low eosinophil proportion). (C) Mild infiltration consisting almost exclusively of eosinophils (low cell density per HPF, high eosinophil proportion). (D) Dense infiltration dominated by eosinophils with fewer lymphocytes and plasma cells (high cell density per HPF, high eosinophil proportion)

For patients with available archived tissue specimens obtained during endoscopic sinus surgery prior to the initiation of dupilumab therapy, a histological workup was conducted at our center. Formalin-fixed, paraffin-embedded samples were sectioned and stained with hematoxylin and eosin (HE). Slides were evaluated under ×400 magnification (high-power field, HPF) by an experienced pathologist. When sufficient tissue was available, two distinct tissue samples per patient were evaluated independently to increase representativeness and accuracy of the inflammatory profile. Tissue eosinophilia was classified based on absolute eosinophil counts per HPF in the most densely infiltrated areas, using the following grading scale according to Lee et al.: Low-grade eosinophilia: ≤10 eosinophils/HPF, Mild-grade eosinophilia: 11–69 eosinophils/HPF, High-grade eosinophilia: ≥70 eosinophils/HPF [27]. Additionally, the relative eosinophil proportion was assessed as the percentage of eosinophils in relation to the total inflammatory cell infiltrate. Examples of high and low eosinophilic tissue infiltration are depicted in Fig. 1. In alignment with established diagnostic frameworks, a proportion of > 20% eosinophils was defined as indicative of tissue eosinophilia based on a proposition by Yang et al. [28].

### Statistical analysis and visualization

Shapiro-Wilk test was employed to test the data for normality. To compare between baseline and individual post-intervention time points paired t-tests were employed for normally distributed data and Mann-Whitney test was performed for not normally distributed data. To examine overall differences across multiple post-intervention time points, the Kruskal-Wallis test was applied. All statistical analyses and data visualization were carried out using SigmaPlotTM 14.5 software tools (Systat Software, San Jose, USA). Unless otherwise indicated results are presented as mean ± standard deviation. A p value of < 0.05 was considered statistically significant. Given the retrospective design of the study, a sensitivity analysis was performed to estimate the minimum detectable between-group difference in the primary outcome measures, assuming a two-sided α level of 0.05 and a statistical power of 80%. To account for multiple comparisons across follow-up time points, post-hoc analyses were adjusted using the Holm–Sidak method. As longitudinal analyses were performed using repeated-measures models, no additional correction across time points was applied to the overall model.

## Results

### Baseline characteristics

A total of 163 patients were included, with a mean age of 49.6 years (range: 21–79); 100 (61.3%) were male. Nearly all patients (98.8%) had undergone at least one ESS, with 62.0% having > 3 ESS and 14.1% >5 ESS. The mean number of surgeries was 2.7, and the mean time since last ESS was 5.8 years.

Type 2 comorbidities were frequent: 79.8% had asthma, 46.6% allergic rhinitis, 45.7% AERD, and smaller percentages had urticaria (0.6%) or atopic dermatitis (3.1%). According to EPOS2020 criteria, 92.6% showed type 2 inflammation, 85.3% needed or could not tolerate OCS, 94.5% reported impaired HRQoL, and 76.1% had significant smell loss. Overall, 93.9% of patients met ≥ 3 EPOS2020 criteria, with 45.6% meeting all five. Detailed baseline characteristics are shown in Table 1.

**Table 1:** Baseline characteristics of the study population

Age at start of therapy	*N* = 163	
	49.6	(21–79)
Sex		
Male	100	(61.3%)
Female	63	(38.7%)
Medical History		
Min 1 ESS	161	(98.8%)
> 3 ESS	101	(62.0%)
> 5 ESS	23	(14.1%)
Time since last ESS (years)	5.8	(0–31)
Mean No. of surgeries	2.7	(0–15)
Evidence of type 2 comorbidities		
Asthma bronchiale	130	(79.8%)
Allergic Rhinitis	76	(46.6%)
Aspirin-Exacerbated Respiratory Disease	64	(45.7%)
Urticaria	1	(0.6%)
Atopic dermatitis	5	(3.1%)
EPOS 2020 criteria		
Evidence of type 2 inflammation	151	(92.6&)
Need for OCS or contraindication	139	(85.3%)
Significantly impaired HRQoL	154	(94.5%)
Significant loss of smell	124	(76.1%)
Diagnosis of comorbid asthma	130	(79.8%)
Number of EPOS2020 criteria met		
1	1	(0.6%)
2	5	(3.1%)
3	19	(11.7%)
4	60	(36.8%)
5	74	(45.6%)
≥3	153	(93.9%)

### Treatment response by demographic and clinical subgroups

Subgroup thresholds were defined according to established clinical and biological parameters frequently applied in CRSwNP research, including the presence of asthma, nasal polyps with NSAID-exacerbated respiratory disease (NERD), allergen sensitization, serum IgE levels, and blood eosinophil counts. These criteria were chosen to explore potential differences in baseline disease profile and treatment response. Group sizes were assessed to ensure adequate statistical power for all comparisons across follow-up time points.

Across all subgroups, dupilumab treatment led to pronounced reductions in NPS and SNOT-22 scores and substantial improvements in SSIT scores over 24 months, with no statistically significant differences between groups at any follow-up (Table 2). Patients with asthma showed a mean NPS reduction of − 4.7 ± 2.1, a SNOT-22 decrease of − 45.5 ± 18.4, and an SSIT improvement of + 4.3 ± 2.8, closely mirroring outcomes in patients without asthma (− 4.0 ± 2.2, − 40.0 ± 20.0, and + 4.1 ± 3.1, respectively). Similarly, patients with NERD achieved NPS, SNOT-22, and SSIT changes of − 4.8 ± 2.4, − 44.9 ± 19.0, and + 4.5 ± 3.1, while those without NERD improved by − 4.6 ± 2.1, − 43.5 ± 18.2, and + 4.3 ± 2.8, respectively. Reductions were also consistent between patients with allergen sensitization (− 4.6 ± 2.0, − 46.0 ± 16.9, + 4.8 ± 2.6) and those without (− 4.7 ± 2.3, − 43.0 ± 18.1, + 3.8 ± 3.0). Comparable patterns were observed between low (< 100 U/L) and high (> 100 U/L) IgE, with NPS changes of − 4.5 ± 2.2 vs. −4.7 ± 2.0, SNOT-22 decreases of − 44.0 ± 17.5 vs. −45.7 ± 18.7, and SSIT gains of + 3.8 ± 3.2 vs. +5.0 ± 2.7. Likewise, patients with blood eosinophil counts < 300 cells/µl improved by − 3.9 ± 3.0 (NPS), − 44.2 ± 20.4 (SNOT-22), and + 3.6 ± 3.2 (SSIT), compared with − 5.1 ± 2.1, − 43.0 ± 17.3, and + 4.9 ± 2.8 in those with higher counts.

**Table 2:** Treatment response by clinical and biological parameters

	*n*	0 months		6 months		12 months		24 months	
	*n*	mean	SD	mean	SD	mean	SD	mean	SD
Age > 45 years	103								
NPS		5.4	1.8	1.5	2.0	1.3	1.9	0.7	1.4
SNOT-22		64.4	15.8	28.4	19.8	23.9	17.2	22.0	16.0
SSIT		4.3	2.9	8.3	2.9	8.3	3.1	9.3	2.8
Age < 45 years	60								
NPS		5.4	1.9	1.4	1.6	1.1	1.4	0.9	2.3
SNOT-22		66.7	14.6	21.9	15.9	22.4	16.7	19.2	16.0
SSIT		3.7	2.5	7.0	2.9	7.3	3.0	7.5	2.8
Female Sex	63								
NPS		5.5	1.9	1.4	1.8	1.2	1.7	0.6	1.1
SNOT-22		68.6	15.4	25.6	21.3	23.5	19.1	20.0	15.2
SSIT		3.4	2.3	7.4	2.9	7.5	3.0	8.4	2.8
Male Sex	100								
NPS		5.3	1.8	1.5	2.0	1.2	1.8	0.9	2.1
SNOT-22		63.3	15.1	26.4	16.9	23.2	15.5	21.3	16.1
SSIT		4.3	2.8	7.4	2.9	7.5	3.0	8.1	3.0
AERD	64								
NPS		5.7	1.8	1.2	1.6	1.0	1.5	0.9	2.5
SNOT-22		67.4	13.7	26.1	19.7	23.7	16.8	22.5	16.1
SSIT		3.3	2.4	7.2	3.2	7.4	3.0	7.8	2.9
No AERD	76								
NPS		5.3	1.8	1.7	2.1	1.4	1.9	0.7	1.3
SNOT-22		62.4	16.5	25.8	18.2	22.3	16.9	18.9	15.4
SSIT		4.4	2.8	7.6	2.7	7.8	3.1	8.7	3.0
Asthma	130								
NPS		5.5	1.8	1.4	1.8	1.2	1.7	0.8	1.9
SNOT-22		66.1	14.6	26.3	18.5	23.4	15.9	20.6	15.1
SSIT		3.8	2.6	7.3	3.0	7.6	3.0	8.1	2.8
No Asthma	33								
NPS		4.9	2.0	1.7	2.2	1.4	1.9	0.9	1.6
SNOT-22		62.0	17.9	25.2	19.6	23.2	20.7	22.0	18.1
SSIT		4.6	3.0	8.0	2.8	7.7	3.1	8.7	3.3
AS	76								
NPS		5.3	1.7	1.2	1.5	1.0	1.2	0.7	2.2
SNOT-22		66.5	15.8	26.3	17.8	23.5	14.8	20.5	15.7
SSIT		3.9	2.7	7.9	2.8	8.1	2.8	8.7	2.5
No AS	87								
NPS		5.5	2.0	1.7	2.2	1.4	2.1	0.8	1.4
SNOT-22		64.2	15.0	25.9	19.4	23 − 1	18.6	21.2	15.9
SSIT		3.9	2.7	7.2	3.1	7.3	3.2	7.7	3.2
Serum IgE ≤ 100 U/l	58								
NPS		5.4	2.0	1.2	1.6	1.0	1.5	0.9	2.4
SNOT-22		63.9	14.0	21.8	15.1	21.3	16.5	19.9	15.2
SSIT		4.1	2.7	7.6	3.1	7.7	3.0	7.9	3.3
Serum IgE > 100 U/l	79								
NPS		5.5	1.7	1.7	2.0	1.4	1.8	0.8	1.4
SNOT-22		67.1	15.6	28.8	20.7	24.6	18.4	21.4	16.5
SSIT		3.7	2.6	7.6	2.8	7.8	3.0	8.7	2.5
B-Eos ≤ 300 cells/µl	26								
NPS		5.3	2.1	1.7	1.9	1.7	1.7	1.4	3.1
SNOT-22		62.5	15.9	28.1	22.2	22.7	21.0	18.3	16.9
SSIT		4.9	3.1	8.3	2.8	7.2	3.5	8.5	3.2
B-Eos > 300 cells/µl	36								
NPS		5.7	1.7	1.2	1.9	1.0	1.8	0.6	1.4
SNOT-22		66.8	15.4	24.5	15.4	24.1	15.8	23.8	15.7
SSIT		3.5	2.4	7.2	3.1	8.1	2.7	8.4	2.9

Notes: Abbreviations: *SD* standard deviation, *NPS* Nasal Polyp Score, *SNOT-22 *Sino-Nasal Outcome Test-22, *SSIT-12* Sniffin’ Sticks 12-item identification test, *AERD* Aspirin-Exacerbated Respiratory Disease, *B-Eos* blood eosinophilia, *AS* allergen sensitization

### Treatment response by histological analysis

**Fig. 2:**
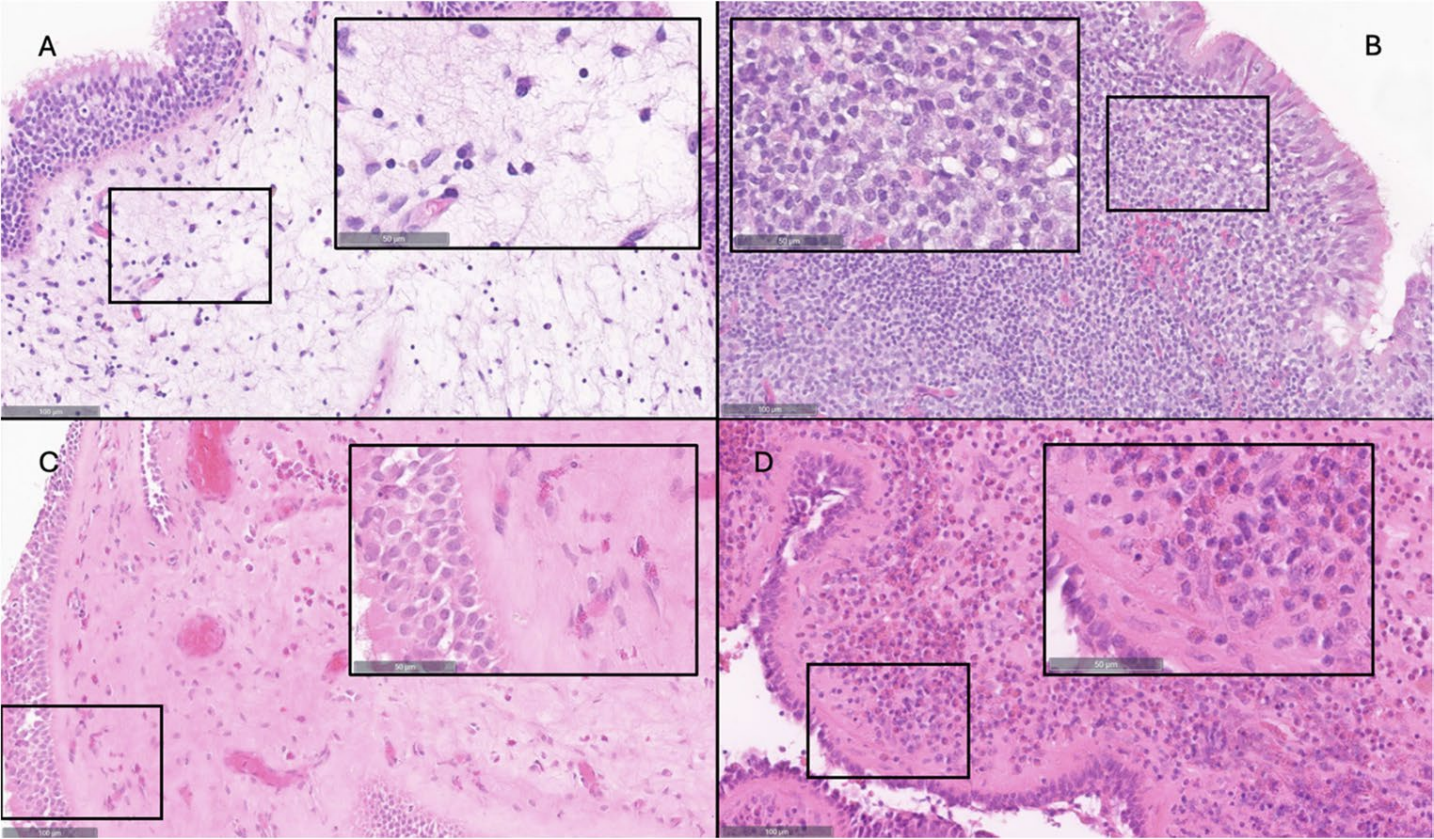
Boxplots displaying the changes of (A) Sinonasal Outcome Test − 22, (B) Bilateral Nasal Polyp Score and (C) olfactory testing displayed on the scale of the Sniffin’ Sticks Identification Test − 12. Dots identifying 5–95% confidence interval. *p < 0.05. Abbreviations: eos, eosinophils; inf, infiltration

Stratification by tissue eosinophilia revealed notable differences in treatment response, while confirming that all subgroups derived substantial benefit from dupilumab therapy (Table 3; Fig. 2). A retrospective re-analysis of archived tissue samples obtained prior to biologic initiation was feasible in 72 patients, allowing systematic assessment of eosinophil infiltration.

**Table 3:** Treatment response by histological analysis

	*n*	0 months		6 months		12 months		24 months	
		mean	SD	mean	SD	mean	SD	mean	SD
≤10 eos/HPF	5								
NPS		5.4	2.3	3.3	2.9	3.6	2.2	3.3	2.9
SNOT-22		59.2	29.1	59.0	28.9	38.4	28.8	27.5	30.0
SSIT		4.6	2.7	5.5	2.3	5.8	2.0	7.0	2.5
11–69 eos/HPF	19								
NPS		5.5	1.2	1.3	2.3	1.1	1.8	0.3	0.6
SNOT-22		67.1	16.6	25.9	16.4	28.5	16.2	22.3	16.5
SSIT		5.5	1.2	6.4	3.0	7.4	2.6	7.8	4.1
≥ 70 eos /HPF	48								
NPS		5.6	1.8	1.1	1.8	1.1	2.0	0.6	1.1
SNOT-22		62.1	15.6	23.4	16.6	22.1	15.6	18.8	15.4
SSIT		3.4	2.5	7.5	3.2	8.0	3.0	7.8	2.9
> 20% eos inf	42								
NPS		5.5	1.8	1.1	1.6	1.0	1.8	0.6	1.1
SNOT-22		61.2	16.7	22.4	15.9	20.8*	15.2	18.6	16.4
SSIT		3.1	2.3	7.5	3.1	8.0	2.8	7.8	3.0
≤ 20% eos inf	30								
NPS		5.6	1.5	1.5	2.6	1.5	2.4	0.8	1.8
SNOT-22		66.2	17.9	31.4	22.3	30.6	19.0	23.3	17.7
SSIT		4.1	2.7	6.5	3.1	7.3	2.9	8.2	3.4

Notes: Abbreviations: *SD *standard deviation, *NPS *Nasal Polyp Score, *SNOT-22* Sino-Nasal Outcome Test-22, *SSIT-12* Sniffin’ Sticks 12-item identification test, *eos* eosinophils, *HPF* high power field, *inf* infiltration

*p < 0,05 vs. 12 months ≤ 20% eos inf

Patients with low eosinophil counts (≤ 10 eos/HPF, *n* = 5) demonstrated only modest improvements over 24 months (NPS − 2.1 ± 2.9, SNOT-22 − 31.7 ± 30.0, SSIT + 2.4 ± 2.5; *p* < 0.001). Due to the small sample size, this cohort was combined with patients with moderate eosinophilia (11–69 eos/HPF, *n* = 19) for subsequent statistical analyses. The combined low–moderate group exhibited significant reductions in NPS and SNOT-22, as well as improvements in SSIT, with outcomes comparable to the high eosinophil group (≥ 70 eos/HPF, *n* = 48; NPS − 5.0 ± 1.8, SNOT-22 − 43.3 ± 15.4, SSIT + 4.4 ± 2.9; *p* < 0.001).

Using relative infiltration thresholds, patients with > 20% eosinophils (*n* = 42) improved by NPS − 4.9 ± 1.8, SNOT-22 − 42.6 ± 16.4, and SSIT + 4.7 ± 3.0 after 24 months (*p* < 0.001), corresponding to a 69.6% reduction in SNOT-22 from baseline. Patients with ≤ 20% infiltration (*n* = 30) showed comparable changes in NPS (–4.8 ± 1.5), SNOT-22 (–42.9 ± 17.7), and SSIT (+ 4.1 ± 3.4), equivalent to a 64.8% SNOT-22 reduction. At 12 months, SNOT-22 scores had decreased by − 40.4 points (–66.0%) in the > 20% group compared with − 35.6 points (–53.8%) in the ≤ 20% group, representing a statistically significant difference in favor of the > 20% group (*p* < 0.044). With the available sample sizes (*n* = 42 vs. *n* = 30), the study had 80% power to detect a minimum between-group difference of 11.4 SNOT-22 points for the 0–12-month change. Smaller subgroup-specific differences may therefore have remained undetected.

Consistently, EUFOREA 2023 criteria indicated higher response rates in the > 20% group (66.6%, 73.8%, and 78.6% at 6, 12, and 18 months) compared with the ≤ 20% group (46.7%, 60.0%, and 70.0%). Again, no patient met criteria for non-response.

## Discussion

In recent years, biologic therapies have fundamentally changed the management of severe, uncontrolled CRSwNP by targeting type 2 inflammation in patients who have often exhausted conventional medical and surgical options [4, 8, 18]. Among these agents, dupilumab has emerged as a first-line biologic in many treatment algorithms due to its consistent efficacy and favorable safety profile demonstrated in randomized controlled trials [24] and real-world studies [14–17, 30, 31]. However, individual responses vary, and identifying reliable predictors of treatment benefit remains a key challenge to optimize outcomes and ensure cost effective use of healthcare resources [21–23].

In this real-world cohort of 163 patients with CRSwNP treated with dupilumab for at least 24 months, we observed substantial and sustained improvements in objective (NPS, SSIT) and patient-reported (SNOT-22) outcomes across all analyzed subgroups. Consistent with previous clinical trial data [24] and observational studies [14–17, 30, 31], dupilumab demonstrated broad efficacy regardless of demographic factors, comorbid asthma, NERD, allergen sensitization, baseline serum IgE levels, or peripheral blood eosinophil counts.

Our findings align with previous reports indicating that serum IgE has limited predictive value for long-term dupilumab response in CRSwNP [22, 32]. While Geba et al. [32] described a non-linear association between baseline IgE and treatment effect in atopic dermatitis—with maximal benefit in patients within an intermediate IgE range—our data did not reveal clinically meaningful differences between low- and high-IgE groups. This supports the view that dupilumab’s efficacy, driven by IL-4/IL-13 pathway blockade, extends across a broad spectrum of IgE phenotypes.

Similarly, in contrast to reports by Habenbacher et al. [23] and Sarnoch et al. [22], we found no evidence that elevated baseline peripheral blood eosinophil count > 0.5 × 10⁹/L predict superior long-term response. Although prior studies linked higher systemic eosinophilia to early gains in polyp scores or SNOT-22, our results suggest that fluctuating peripheral markers do not reliably capture long-term disease control. Potential explanations include differences in follow-up duration, patient selection, or the possibility that local tissue eosinophilia—rather than fluctuating systemic counts—plays a more decisive role in long-term disease control.

Subgroup analyses further supported previous findings from CHRINOSOR [30] and related studies [31], showing that prior surgical history, allergen sensitization, and demographic factors were not predictive of treatment response. This highlights the broad applicability of dupilumab for guideline-selected CRSwNP patients regardless of these baseline characteristics.

Tissue eosinophilia is widely regarded as a robust marker of type 2 inflammation in CRSwNP, closely linked to local cytokine expression and considered a more reliable histopathological biomarker than clinical surrogates [29–31]. Prior studies indicate that tissue eosinophilia correlates more closely with disease severity in CRSwNP than peripheral blood eosinophil counts, which show only a moderate association with local inflammation [32]. In routine practice, however, pathology reports rarely include quantitative eosinophil counts per high-power field (HPF), often limiting assessments to qualitative descriptions such as “eosinophil-rich inflammation.” To explore its prognostic relevance, we retrospectively re-evaluated archived polyp tissue from all eligible patients. Elevated tissue eosinophil counts represent a hallmark of type 2 inflammation, which constitutes the primary pathway targeted by dupilumab.

Using established thresholds (Yang et al.: >20% eosinophils [28]; Lee et al.: ≥70 eosinophils/HPF [27]), we found that patients with > 20% eosinophils had significantly greater SNOT-22 reductions at 12 months compared with those with ≤ 20%. While this difference attenuated by 24 months, trends toward superior outcomes persisted in the high-grade eosinophilia group, including greater reductions in NPS and improved SSIT scores. These findings are consistent with recent real-world data linking higher baseline tissue eosinophilia to greater symptomatic improvement with dupilumab. However, these data were limited to a 12-month follow-up and applied eosinophil thresholds that are not uniformly standardized, limiting direct comparability [33].

Importantly, classification of treatment response according to the EUFOREA 2023 criteria demonstrated parallel results: high eosinophil groups consistently achieved higher response rates across follow-up compared with mild and moderate groups, while no patient met criteria for non-response.

Collectively, these findings suggest that elevated tissue eosinophilia accelerates and amplifies early clinical gains, with patients in the highest strata achieving the most pronounced improvements. Nevertheless, long-term benefits of dupilumab remained substantial across all eosinophil categories, underscoring both the prognostic value of tissue eosinophil quantification and the broad applicability of treatment in CRSwNP.

This study has several limitations that should be considered when interpreting the findings. First, its retrospective, single-center design may restrict generalizability to broader CRSwNP populations, particularly across healthcare systems with different referral patterns or biologic prescribing criteria. Second, our focus on long-term outcomes may have reduced sensitivity for detecting early predictors of short-term response. Third, quantitative histological analysis was limited to of patients with available archived tissue samples, introducing potential selection bias. Moreover, standard pathology workflows typically do not include eosinophil counts per HPF, and while we retrospectively applied the Yang and Lee thresholds, these criteria have not yet been universally validated for clinical decision-making. Finally, peripheral biomarkers such as blood eosinophils and serum IgE can fluctuate over time and may be influenced by concurrent treatments, potentially diluting their predictive value in long-term analyses.

## Conclusion

In this long-term, real-world analysis of dupilumab therapy for CRSwNP, higher levels of tissue eosinophilic infiltration—particularly exceeding the > 20% threshold—were associated with greater and more sustained improvements in patient-reported outcomes. By contrast, systemic biomarkers such as baseline blood eosinophil count and serum IgE did not predict long-term benefit. These findings highlight the prognostic value of quantitative histopathology and suggest that incorporating eosinophil counts into routine pathology workflows could support more precise stratification of patients eligible for biologic therapy. Dupilumab, however, provided durable benefit across all patient groups, reinforcing its broad applicability as an effective treatment for uncontrolled CRSwNP.
